# Practice of Menstrual Hygiene and Associated Factors among Adolescent School Girls in Dang District, Nepal

**DOI:** 10.1155/2020/1292070

**Published:** 2020-07-24

**Authors:** Chet Kant Bhusal

**Affiliations:** Department of Community Medicine, Universal College of Medical Science and Teaching Hospital, Tribhuvan University, Bhairahawa, Rupandehi, Nepal

## Abstract

**Background:**

Menstrual hygiene management has not been sufficiently addressed in developing countries. In many Nepalese societies, menstrual practices are still surrounded by sociocultural restrictions and taboos resulting in adverse health outcomes for adolescent girls. The purpose of this study was to determine menstrual hygiene practice and sociodemographic as well as socioeconomic factors associated with good menstrual hygiene practice amongst adolescent school girls in Dang district, Nepal.

**Methods:**

A cross-sectional study was conducted in Dang district, Nepal, among 406 adolescent girls studying in grades 8, 9, and 10 between ages of 10 and 19 years from April to October 2019. Randomly 5 units were selected from a total of 10 local units. After 5 units had been decided, 10 schools consisting of 5 government and 5 private schools were selected through a disproportionate stratified random sampling technique. A further 406 students were then selected randomly from the 10 selected schools. Bivariate analysis was used primarily to assess the association between dependent and independent variables and final measure of association was odds ratio. Variables which were associated with bivariate analysis were entered into a multivariable logistic regression model to identify associated factors of menstrual hygiene practice.

**Results:**

The mean age and family size were 15.13 ± 1.19 and 5.58 ± 1.81, respectively. A total of 272 (67.0%) adolescents have good menstrual hygiene practice. Mothers and fathers with literature educational background (adjusted odds ratio = 0.52, confidence interval: 0.30–0.89 and AOR = 2.55, CI: 1.26–5.15, respectively), family size greater than or equal to 5 (AOR = 0.61, CI: 0.37–0.98), and living with relatives (AOR = 0.45, CI: 0.24–0.85) were significantly associated with good menstrual hygiene practice.

**Conclusions:**

Educational status of mother and father, family size, and living status were found to be independent associated factors of menstrual hygiene practice. In this context, this study demonstrates that administrators and policy makers should provide specific education regarding menstrual hygiene to both parents. Similarly local government needs to subsidize hygiene towels for school adolescents.

## 1. Introduction

Onset of menstruation is one of the vital changes happening in all females during their period of adolescence [1]. Menarche is not just a physiological process but it is a psychological, social, and behavioral transition from adolescence to womanhood [2]. Menstrual hygiene means necessities and requirements such as the use of sanitary pads or clean and soft absorbents, adequate washing of the genital area, proper disposal of used absorbents, and other special healthcare needs of women during monthly menstrual cycle [3, 4]. In woman's life, good hygiene practice during menstruation is very important which prevents from adverse health outcomes [5, 6].

Though menstruation is a normal physiological process, it is still surrounded with social taboos, supernatural beliefs, misconceptions, and malpractices, which is very challenging for girls in developing countries [7, 8]. Due to these social stigmatic, cultural, and religious restrictions, menstrual practices are regarded as big limitation for menstrual hygiene management [9]. Menstrual hygiene is very important; however it is still a neglected area of concern in many parts of the world [2]. Despite the fact that menstruation is a normal physiological process in females, it is not easy for every adolescent girl to maintain good hygiene [10]. Due to the lack of prior information about menstruation, girls experience different feelings such as fear, embarrassment, and guiltiness during their vaginal bleeding [11]. Adolescent girls particularly in developing countries still lack information about good menstrual hygiene practices [6]. Management of menstrual hygiene is considered as taboos in many parts of Nepal. Traditional and supernatural beliefs regarding menstruation may have negative impact on self-respect, health, and education of adolescent girls [12]. Several studies have reported that infections may occur due to lack of hygiene during menstruation [1, 13]. If menstrual period is not properly handled and safe hygiene is not practiced, this may lead to poor quality of life resulting from distress, reproductive tract infection, genitourinary tract infections, smelling, guiltiness, cervical cancer, poor academic performance, and school dropout [7, 14, 15]. Poor menstrual practice is also connected with many other complications such as premature births, stillbirths, miscarriages, infertility problems, and carcinoma of cervix [11]. Taboos and misconceptions on the subject of menstruating girls and menstrual hygiene develop in gender inequality and degradation of women empowerment [16]. Menstruation is regarded as a taboo even by the teachers; therefore, they do not offer information and guidance on the importance and management of menses. In developing countries, adolescent girls face various menstrual hygiene management challenges, especially at school [17].

Although there has been a growing literature on various aspects of menstrual hygiene, limited available studies in Nepal had focus on finding association between good menstrual hygiene practice and social factors among adolescent school girls. This is a hidden and unaddressed public health issue in country settings of Nepal. Therefore, this study was mainly conducted to identify menstrual hygiene practice and sociodemographic as well as socioeconomic factors associated with good menstrual hygiene practice amongst adolescent school girls in Dang district, Nepal. The result of this study might provide valuable information for health service providers, administrators, and policy makers in developing appropriate intervention programs. Moreover, the findings of this study will be used as baseline for further researcher.

## 2. Materials and Methods

### 2.1. Study Design and Source of Population

A cross-sectional study was conducted in Dang district, Nepal, from April to October 2019. School-going adolescent girls both from government and private schools studying in grades 8, 9, and 10 of age group 10–19 were included in the study. Similarly, girls whose menarche has not started, who had severe mental problems, and who were absent during the day of data collection were set as exclusion criteria.

### 2.2. Sample Size Determination and Sampling Technique

Sample size was calculated using formula *N* = *Z*^2^*pq*/*L*^2^ [18] with 95% level of confidence interval, and 6% margin of error; and 39.9% of respondents had practiced good menstrual hygiene [19]. Initial sample size of 256 was multiplied by design effect of 1.5 as multistage stratified probability random sampling was used. Thus sample size was 384. Considering 5.5% nonresponse rate, the final sample size was 406. A multistage probability random sampling was used as sampling technique among a total of 142 secondary schools of Dang district, Nepal. Among a total of 10 local units, 5 were selected randomly. Then, from selected 5 units, 10 schools consisting of 5 government and 5 private schools were selected by disproportionate stratified random sampling technique through nonreplacement lottery method. Further, 406 students were selected randomly from 10 schools which consisted of 41 from each government and 40 from each private school. Since sample size was 406, one additional student was taken from private school.

### 2.3. Data Collection Procedures and Validity

Data were collected using semistructured questionnaire by applying self-administered interview technique. Questionnaire was translated into Nepali and again retranslated into English language to find misinterpretation. The questionnaire was pretested among 10% of total sample residing in Bhairahawa, Rupandehi. Both English and Nepali version questionnaires were made and used according to familiarity of students. Two-day training was provided to three data collectors having qualification of Master's in Information Communication Technology, Bachelor in Public Health, and Bachelor of Science in Agriculture. All the filled questionnaires were reviewed and checked by the principal investigator on regular basis.

### 2.4. Data Processing and Analysis

Data were entered into Microsoft Excel and exported to Statistical Package for Social Science (SPSS) software version 20 for analysis. Characteristics of the sample were described using mean and standard deviation. Bivariate analysis was used primarily to assess the association between dependent and independent variables and those variables found to be associated with bivariate analysis (*p* < 0.05) were entered into the multivariate logistic regression model (stepwise backward likelihood ratio method) to identify the associated factors of practice of menstrual hygiene.

### 2.5. Setting

Dang district is located in inner Terai and mid hills of Rapti zone in the mid-western development region of Nepal. There are 2 submetropolitan cities, 1 municipality, and 7 rural municipalities in the district. The district has total population of 552,583 and annual population growth rate of 1.78 [20]. There are altogether 142 secondary schools including 86 private and 56 government; similarly there are total 24,622 students in secondary level in Dang district which includes 12,905 female and 11,717 male students [21].

### 2.6. Measurement of Practice on Menstrual Hygiene

The students' menstrual hygiene practice was assessed using 10 practice specific questions. Each correct response scored one point, whereas wrong or do not know answer did not get score, and accordingly sum score was calculated out of 10 points as similarly described in previous studies [7]. The mean score was 7.07; therefore participants with a total score less than 7 (0–6) were considered as having poor practice while those who scored 7–10 points were considered as having good practices.

### 2.7. Ethics Approval and Consent to Participate

Ethical approval was obtained from of Universal College of Medical Science and Teaching Hospital Institutional Review Committee (UCMS/IRC/063/19). Concerned stakeholders were officially contacted with letters and permission was obtained at all levels. The study was explained to participants. Since most of the respondents were below age group 18 years, verbal consent was obtained from children's parents/guardians through telephone and after coordinating with parents, written informed consent was taken from school teachers before interview.

## 3. Results

The mean age and family size were 15.13 ± 1.19 and 5.58 ± 1.81, respectively. Out of total 406 school adolescents, more than half (55.7%) live in submetropolitan city and municipalities. Majority (91.6%) of the adolescents followed Hindu religion. Two-thirds (67.0%) of respondents had nuclear family. Nearly one-fourth (23.6%) of the respondents' mothers were unable to read and write. Slightly less than half (47.5%) of the mothers were homemakers. More than three-fourths (78.3%) of respondents' fathers were engaged in occupations other than agriculture for their livelihood (Table 1).

Most of the girls, 363 (89.4%), had used some form of absorbents during their menstruation. Nearly half (45.1%) of the respondents did not dry washed reusable clothes in direct sunlight, most, 83.7%, of the girls disposed used pads in dustbins or pit latrine. Only 16.5% of the school girls took daily bath during menstruation (Table 2).

**Figure 1:**
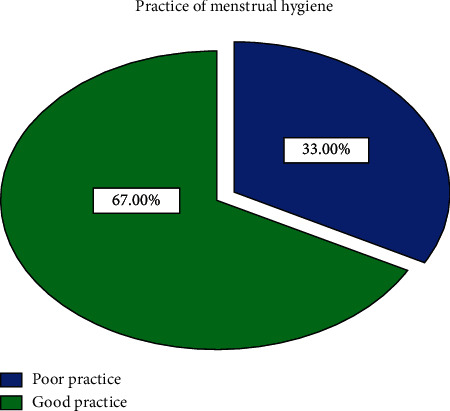
Distribution of adolescent school girls' practice grading on menstruation hygiene.

The mean score of school girls' practice of menstruation and its hygienic management was 7.07 ± 1.84; therefore participants with a total score less than 7 (0–6) were considered as having poor practice while those who scored 7–10 points were considered as having good practices. Concerning the practice level of school-going adolescent girls, among 406 girls, two-thirds (272 (67.0%)) practiced good menstrual hygiene whereas one-third (134 (33.0%)) practiced poor menstrual hygiene (Figure 1).

Table 3 showed socioeconomic and sociodemographic factors associated with practice of menstrual hygiene. Those variables, which were found statistically significant with *p* value lesser than or equal to 0.05 in bivariate analysis, were entered into the multivariate regression analysis model which identified mother's education, father's education, and size of family as associated factors with menstrual hygiene practice. Adolescent girls whose mothers were literate were 0.52 times less likely (AOR = 0.52, CI = 0.30–0.88) to have good practices regarding menstrual hygiene and its management. However, the odds of having good menstrual hygiene practice were high among girls whose fathers were from literate backgrounds (AOR = 2.61, CI = 1.31–5.23) and less among those who have 5 or more members in the family (AOR = 0.61, CI = 0.37–0.98) (Table 3).

## 4. Discussion

Literate mothers, literate fathers, size of family, and living with parents were associated factors of good menstrual hygiene practice in this study. The group of girls having 1 to 4 family members have good menstrual hygiene practice; this might be due to more interaction and communication with mothers in small families. More than three-fourths of school girls in developing countries like Nepal change absorbent materials in their school too. Less than one-fifth of the school girls took daily bath during menstruation. In this study, about two-thirds of school-going adolescent girls practiced good menstrual hygiene which is in line with studies done in Nepal [22] and Ethiopia [23]. However, several studies done in Ethiopia [24], Nigeria [25], and Ghana [26] found that higher proportion of the girls practiced good menstrual hygiene as compared to this study. In contrast to this study, small portion of good menstrual hygiene practice was also observed in several studies such as studies done in Nepal [12], Ethiopia [6, 19], and Nigeria [27]. This disparity might be due to different study setting, change in time, and divergence scoring system for measuring the practice level of menstrual hygiene in different studies.

Majority of adolescent girls in the study area used some form of the absorbents during their menstruation which is in line with previous study conducted in Ethiopia where 90.98% of school girls used some forms (sanitary pad and homemade clothes) during their menstrual period [8]. More than two-thirds of girls used disposable sanitary pads during their menstruation which is in concordance with the previous studies done in Nepal [28], India [29, 30], and Egypt [31]. However, several previous studies done in rural area of Nepal [32] and India [3, 33, 34] found that small portion of adolescent girls used sanitary pads during their menstruation. This might be due to the advancement in time and different study setting. This study revealed that more than half of the girls cleaned their genital organ with clean water during their menstruation; this is supported by the study done in Nepal [12] and India [35]. Less than one-fifth of adolescent girls in the current study took bath daily during menstruation which is supported by the study done in Nepal [36] where only 14.3% of girls took bath more than 3 times in one menstrual period. Nearly two-thirds of the adolescent girls in this study cleaned their genital organ at least thrice a day; however, a study done in Amhara regional state, Ethiopia, found that smaller portion of the girls washed their genitalia at least three times per day [24]. On the top of that, another study done in Nagpur district, India, found that only one-third of girls cleaned their external genitalia two times or less a day [33]. The present study revealed that three-fifths of school girls practiced changing of absorbent materials at least three times a day which is in accordance with the study done in Uganda [37]. However, several other studies reported dissimilar result; one study done in rural area of Nepal found that around two-fifths of similar school girls changed absorbents at least three times per day [32] and another study done in Sokoto, Nigeria, found that higher portion (70%) of the girls changed absorbents at least three times per day [38]. This might be due to time and focusing of rural area by previous study done in Nepal. In this study, more than three-fourths of respondent girls used to change absorbent materials in school; however, it is inconsistent with previous study conducted in Nagpur district of India where lower percentage of girls changed pads at school [33]. More than half of the adolescent girls in this study are drying washed reusable clothes in direct sunlight which is in line with the previous studies conducted in India [33] and Nigeria [39]. The present study reported that majority of the respondents disposed used menstrual materials in dustbins or pit latrine toilets which is accordance with the study done in Kathmandu valley of Nepal [40]. The majority of the girls in this study used to take bath with soap and water during their menstrual period which is in concordance with the study done in Northeast Ethiopia [8].

In the current study, mother's education was significantly associated with practice regarding menstrual hygiene which is also supported by several studies such as study done in India [39, 41], Adama Town, Ethiopia [6], Western Ethiopia [19], and Nigeria [38]. The study found significant association of practice of menstrual hygiene with father's education. Similar result was found in the bivariate analysis of another study done in rural area of West Bengal, India [41]. However, in contrast to this study, father's education was not found significant with practice of menstrual hygiene in another study done in India [42]. Positive association was found between good menstrual hygiene and size of family. The girls having 1 to 4 family members have good menstrual hygiene practice. There was positive association between good hygienic practice and living with parents which is in line with another study done in Nigeria [27]. This might reflect that girls who live with their parents discussed openly; they were better observed and guided during their menstrual period compared to those who reside with relatives.

## 5. Limitations of the Study

Studies from different parts of Nepal observed mean age of menarche as 13 years. As this study was conducted among adolescent girls studying in grades 8 to 10, this might miss some young girls who are studying in lesser class and have just started their menarche. Therefore, further studies are recommended without excluding those younger girls.

## 6. Conclusions

One-third of the school-going adolescent girls practiced good menstrual hygiene. The main contributors of this are mother's education, father's education, family size, and living status of children. In this context, administrators and policy makers were recommended to provide specific education on menstrual hygiene to both parents. Similarly, local government needs to subsidize hygiene towels for school adolescents.

## Figures and Tables

**Table 1:** Distribution of background related characteristics of study population.

General characteristics	Frequency (*n* = 406)	Percentage
Age		
10–13 years	34	8.4
>13 Years	372	91.6
Mean age ± SD; 15.13 ± 1.19		

Residence		
Cities (submetropolitan city and municipalities)	226	55.7
Village (rural municipalities)	180	44.3

Religion		
Hindu	372	91.6
Non-Hindu (Christian and Muslim)	34	8.4

Family type		
Nuclear	272	67.0
Joint and extended	134	33.0

Family size		
1 to 4	120	29.6
5 and above	286	70.4

Type of school		
Government	205	50.5
Private	201	49.5

Education of mother		
Illiterate	96	23.6
Literate	310	76.4

Education of father		
Illiterate	37	9.1
Literate	369	90.9

Occupation of mother		
Homemaker	193	47.5
Other than homemaker	213	52.5

Occupation of father		
Agriculture	88	21.7
Other than agriculture	318	78.3

**Table 2:** Distribution of practice of adolescent school girls about menstruation hygiene and its management.

Practice about menstruation and its management	Number (*n* = 406)	Percentage
Used some form of absorbents during menstruation	363	89.4
Used sanitary pads during menstruation	293	72.2
Cleaning of genital organ with clean water	225	55.4
Daily bath during menstruation	67	16.5
Cleaning of genital organ at least three times a day	260	64.0
Changing of absorbent materials at least three times a day	241	59.4
Changing of absorbent materials in school	318	78.3
Drying of washed reusable clothes in direct sunlight	223	54.9
Disposed used pads in dustbins or toilets	340	83.7
Bathing with soap and water during menstruation	343	84.5

**Table 3:** Factors associated with level of practice in using bivariate and multivariate analysis.

Characteristics	Practice level (%)		*p* value	^a^COR 95% CI	^b^AOR 95% CI
	Poor practice	Good practice			
Mother's education					
Illiterate	23 (24.0)	73(76.0)	0.031^*∗*^	1	1
Literate	111(35.8)	199(64.2)		**0.57(0.33–0.95)**	**0. 52(0.30-0.89)**

Father's education					
Illiterate	20(54.1)	17(45.9)	0.004^*∗*^	1	1
Literate	114(30.9)	225(69.1)		**2.63(1.33–5.21)**	**2.55(1.26–5.15)**

Father's occupation					
Agriculture	27(30.7)	61(69.3)	0.601	1	-
Other than agriculture	107(33.6)	211(66.4)		0.87 (0.53–1.45)	

Size of family					
1 to 4 members	31(25.8)	89(74.2)	0.047^*∗*^	1	1
5 and above	103(36.0)	183(64.0)		**0.62(0.38–0.99)**	**0.61(0.37–0.98)**

Live with					
Parents	111(30.8)	249(69.2)		1	1
Relatives	23(50.0%)	23(50.0%)	0.009^*∗*^	**0.89(0.57–1.37)**	**0.45(0.24–0.85)**

^*∗*^Significant at *p* < 0.05, 1 = reference category, ^a^crude odds ratio, and ^b^adjusted odds ratio.

## Data Availability

The raw data under identification policy will be provided upon request through e-mail to corresponding author.

## Abbreviations

AOR: Adjusted odds ratio
CI: Confidence interval
OR: Odds ratio
SD: Standard deviation
SLC: School leaving certificate
SPSS: Statistical package for the social sciences.
